# High-fat diet plus HNF1A variant promotes polyps by activating **β**-catenin in early-onset colorectal cancer

**DOI:** 10.1172/jci.insight.167163

**Published:** 2023-07-10

**Authors:** Heyu Song, Ricky A. Sontz, Matthew J. Vance, Julia M. Morris, Sulaiman Sheriff, Songli Zhu, Suzann Duan, Jiping Zeng, Erika Koeppe, Ritu Pandey, Curtis A. Thorne, Elena M. Stoffel, Juanita L. Merchant

**Affiliations:** 1 Department of Medicine, Division of Gastroenterology and Hepatology, Arizona Comprehensive Cancer Center, and; 2 Department of Cellular and Molecular Medicine, University of Arizona, Tucson, Arizona, USA.; 3 Human Biology Division, Fred Hutchinson Cancer Center, Seattle, Washington, USA.; 4 Department of Urology, University of Arizona College of Medicine, Tucson, Arizona, USA.; 5 Michigan Medicine Cancer Genetics Clinic and; 6 Department of Internal Medicine, University of Michigan, Ann Arbor, Michigan, USA.

**Keywords:** Gastroenterology, Genetics, Cancer, Colorectal cancer, Genetic variation

## Abstract

The incidence of early-onset colorectal cancer (EO-CRC) is rising and is poorly understood. Lifestyle factors and altered genetic background possibly contribute. Here, we performed targeted exon sequencing of archived leukocyte DNA from 158 EO-CRC participants, which identified a missense mutation at p.A98V within the proximal DNA binding domain of Hepatic Nuclear Factor 1 α (HNF1A^A98V^, rs1800574). The HNF1A^A98V^ exhibited reduced DNA binding. To test function, the HNF1A variant was introduced into the mouse genome by CRISPR/Cas9, and the mice were placed on either a high-fat diet (HFD) or high-sugar diet (HSD). Only 1% of the HNF1A mutant mice developed polyps on normal chow; however, 19% and 3% developed polyps on the HFD and HSD, respectively. RNA-Seq revealed an increase in metabolic, immune, lipid biogenesis genes, and Wnt/β-catenin signaling components in the HNF1A mutant relative to the WT mice. Mouse polyps and colon cancers from participants carrying the HNF1A^A98V^ variant exhibited reduced CDX2 and elevated β-catenin proteins. We further demonstrated decreased occupancy of HNF1A^A98V^ at the *Cdx2* locus and reduced *Cdx2* promoter activity compared with WT HNF1A. Collectively, our study shows that the HNF1A^A98V^ variant plus a HFD promotes the formation of colonic polyps by activating β-catenin via decreasing *Cdx2* expression.

## Introduction

Colorectal cancer (CRC) is the third most common cancer diagnosed in both men and women in the United States. In 2022, it is estimated that ~150,000 new cases of CRC will be diagnosed, causing about 52,580 deaths (1). Despite increased screening and lifestyle modifications, there remains a 2% increase each year in the incidence of people younger than 50 who develop CRC, representing renewed interest in understanding what is accelerating disease pathogenesis (2).

Early-onset CRC (EO-CRC) is defined as CRC diagnosed at age < 50 and possesses unique clinical, genetic, and epigenetic characteristics (3, 4). According to the National Cancer Database, EO-CRC consists of 13.3% of all CRC (5). While 20% of EO-CRC exhibit hereditary mutations, the majority are sporadic (6). The etiology of sporadic EO-CRC is multifactorial and is likely derived from the interaction between genetic variants and environmental risk factors. For instance, obesity, diabetes, smoking, and alcohol are well-studied lifestyle factors that associate with EO-CRC. Furthermore, dietary factors such as a high-fat diet (HFD) strongly correlates with the development of CRC (7, 8). Moreover, the goal of emerging studies has been to identify impactful novel genetic polymorphisms in individuals with EO-CRC. Although a recent meta-analysis identified 160 SNPs in 96 distinct genes associated with a predisposition to nonhereditary CRC (9), few studies have investigated how environmental factors interact with the genetic landscape leading to CRC development.

Here we identified a germline missense mutation at p.A98V within the proximal DNA binding domain of Hepatic Nuclear Factor 1 α (*HNF1A^A98V^*, rs1800574) by performing targeted exon sequencing of archived leukocyte DNA from EO-CRC individuals. The current understanding of HNF1A function has emerged primarily from studies of this transcription factor in the context of mature-onset diabetes of the young 3 (MODY3) and the risk for diabetes mellitus (DM). HNF1A, also known as T cell factor 1 (TCF1), encodes a transcriptional regulatory protein that is required for the expression of several liver specific genes (10). It functions as a homodimer and binds to the inverted palindrome 5′-GTTAATNATTAAC-3′. The presence of mutations in the *HNF1A* gene is one of the most common causes of MODY3 (11). In addition to its association with increased type 2 DM (T2DM) risk, the *HNF1A^A98V^* variant is also linked to dyslipidemia (12, 13). Given its role in insulin resistance and lipid metabolism, we sought to investigate how diet interacts with the altered genetic background in the development of EO-CRC. We found that the *HNF1A*^A98V^ variant exhibits impaired DNA binding and induced colon polyp formation in genetically engineered mice when exposed to a HFD. Furthermore, our data suggest that reduced HNF1A function precluded CDX2 suppression of β-catenin and created a permissive genetic landscape for polyp formation.

## Results

### HNF1A^A98V^ variant identified in EO-CRC.

**Figure 1:**
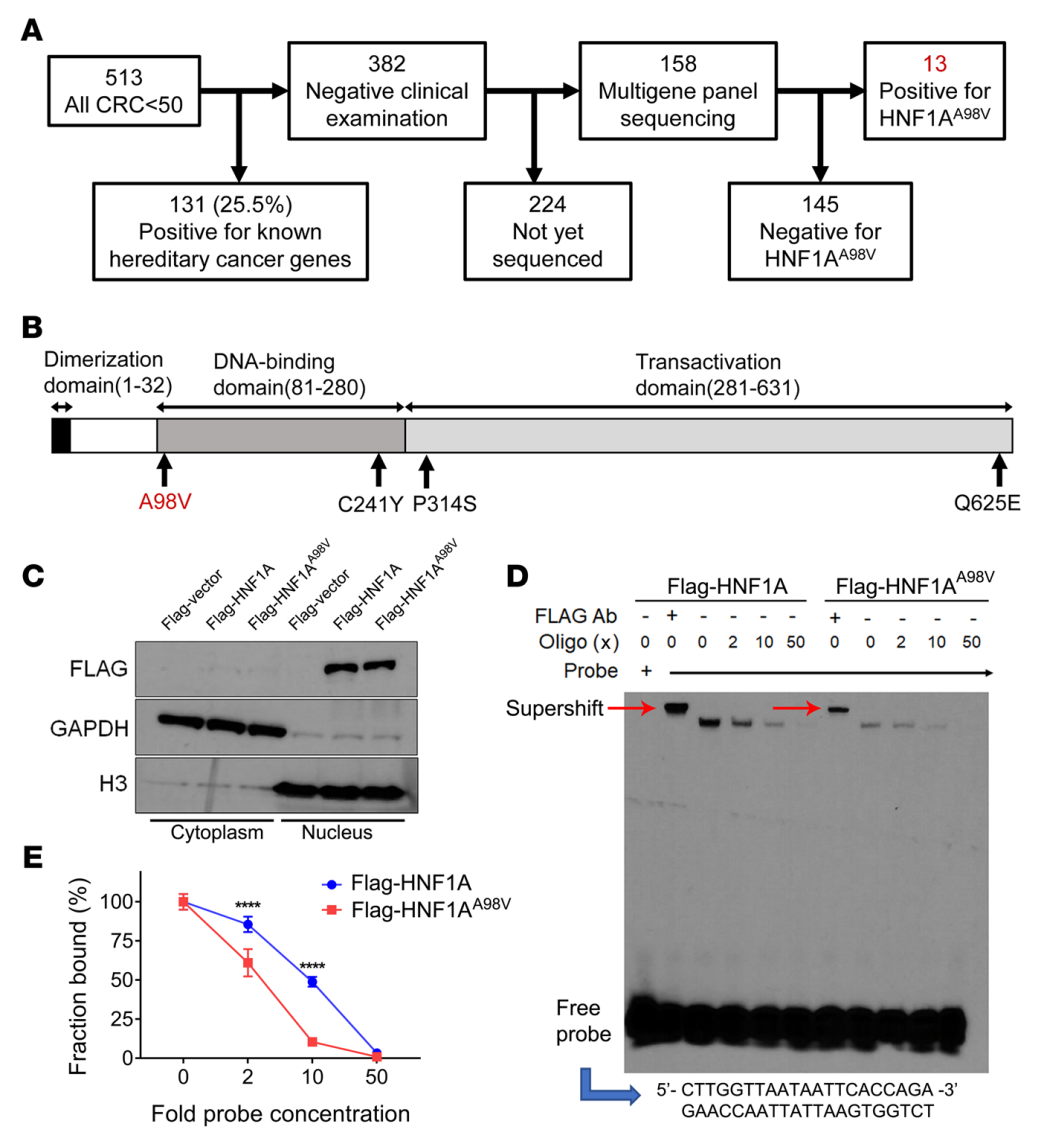
HNF1A^A98V^ was identified in EO-CRC and demonstrated reduced DNA binding affinity and avidity. (**A**) Flow diagram of exon sequencing cancer gene panel from Qiagen of leukocytes from participants at the University of Michigan (UM) GI Genetics Clinic. (**B**) Location of HNF1A missense mutations identified in EO-CRC participants with 13 participants having a missense mutation at A98V located within the DNA binding domain. (**C**) Flag-tagged HNF1A and HNF1A^A98V^ variant plasmids were transfected into HCT116 cells, followed by cellular fractionation. (**D**) The DNA binding of HNF1A and HNF1A^A98V^ proteins was evaluated by EMSA. FLAG antibody was added to confirm protein identity by a supershift (red arrow). The unlabeled probe was added to the reaction at 2, 10, and 50 times the probe concentration. (**E**) The percentage of probe shifted per total probe added was plotted as a function of unlabeled probe for HNF1A and the A98V variant and showed lower avidity for the A98V variant. Five individual experiments were performed. Data represent mean ± SEM. 2-way ANOVA, ****P < 0.0001.

To identify novel germline mutations in EO-CRC, 513 patients who presented with CRC prior to the age of 50 years were identified in the University of Michigan Cancer Genetic Clinic Database. After excluding those who had known hereditary germline gene mutations (*n* = 131), 158 of the remaining 382 samples were sequenced using the Qiagen NextGen sequencing Multigene Cancer Panel (Figure 1A). Sixteen missense mutations were identified in *HNF1A*, the MODY3 locus. The missense mutation (rs1800574) converts alanine to valine at amino acid residue 98 and was identified in 13 of the 16 cases; while the remaining 3 mutations were located at C241Y, P341S, and Q625E (Figure 1B). HNF1A protein contains 3 functional domains: an N-terminal dimerization domain (residues 1–32), a bipartite DNA binding domain (residues 81–280) (14), and a C-terminal transactivation domain (residues 281–631) (15).

Given the 4-fold higher frequency of this gene variant compared with its expression in the general population (2%), we analyzed the function of this variant. The patient demographics summarized in Table 1 and Supplemental Table 1 (supplemental material available online with this article; https://doi.org/10.1172/jci.insight.167163DS1) show that 15% of the participants had diabetes, while at least 30% had dyslipidemia or were obese.

### HNF1A^A98V^ reduces DNA binding.

Since the A98V variant was located in the HNF1A DNA binding domain, we examined whether this missense mutation affected its ability to bind DNA. Flag-tagged *HNF1A* and *HNF1A^A98V^* variant expression vectors were transfected into the HCT116 colon cancer cell line, which expresses minimal endogenous HNF1A protein. After 2 days, nuclear fractions were prepared (Figure 1C; See complete unedited blots in the supplemental material) and incubated with the *HNF1A* consensus DNA binding site in electrophoretic mobility shift assays (EMSAs) (Figure 1D). An unlabeled probe was used to compete for protein binding and showed that, while both HNF1A and the HNF1A^A98V^ recognized the DNA binding site, HNF1A^A98V^ exhibited lower affinity for the site (Figure 1, D and E). Flag antibody supershifted both complexes, confirming protein identities. Taken together, these results show that the HNF1A^A98V^ variant is a loss-of-function mutant that exhibits reduced DNA binding affinity and avidity.

### HNF1A^A98V^ increases susceptibility to colon polyps in mice on a high-fat diet.

**Figure 2:**
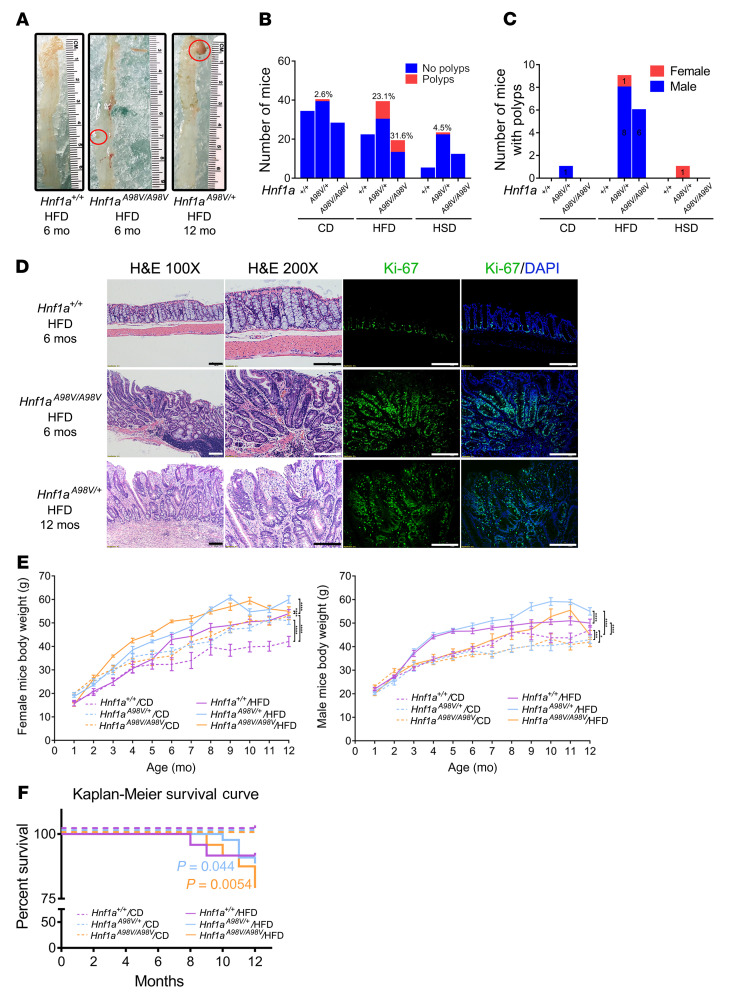
HNF1A*^A98V^* conferred susceptibility to polyp formation in mice with a high-fat diet. (**A**) Macroscopic images of colons from *Hnf1a^+/+^,* *Hnf1a^A98V/+^*, and *Hnf1a^A98v/A98V^* mice. Polyps were identified in the *HNF1A^A98V^* genotypes mice as indicated. The corresponding microscopic images of these polyps were shown in **D**. (**B**) Bar graph showing the total number of mice and the number and percentage of mice with polyps in each group (red). Note that mice were euthanized at different ages to examine polyp formation. The details were included in Supplemental Table 2. (**C**) Bar graph showing the number of male and female mice with polyps. (**D**) Representative H&E and IF staining for Ki-67 (green); DAPI (blue). Scale bar: 100 μm. (**E**) Monthly mouse weights are plotted for each genotype. Two-way ANOVA; *P < 0.05; ***P < 0.001; ****P < 0.0001. (**F**) Percent survival for each mouse on CD versus a HFD per genotype is shown in Kaplan-Meier survival curves.

Given that the *HNF1A^A98V^* variant represented a single-nucleotide mutation resulting in a conservative amino acid change, we tested its function in vivo by introducing the variant into the mouse genome by CRISPER/Cas9 technology on a C57BL/6J genetic background. Since HNF1A is involved in glucose and lipid metabolism and essentially no polyps developed in the *Hnf1a^A98V^* mice on normal mouse chow, after weaning, *Hnf1a^+/+^*, *Hnf1a^A98V/+^*, and *Hnf1a^A98V/A98V^* mice were placed on 3 diets —normal mouse chow (control diet, CD), a HFD, and a high-sugar/fructose diet (HSD). All mice had free access to normal water, and the mice were followed for 12 months. Both the *Hnf1a^A98V/+^* and *Hnf1a^A98V/A98V^* mice developed polyps by 6 months of age (Figure 2A and Supplemental Table 2). By contrast, polyps were rarely found in the *Hnf1a^A98V/+^* and *Hnf1a^A98V/A98V^* mice on normal chow (<1%) or HSD (<3%) (Figure 2B and Supplemental Table 2). Since prior epidemiological studies have identified low-fiber and HFDs as risk factors for CRC (16), the *Hnf1a^A98V/+^* and *Hnf1a^A98V/A98V^* mice were placed on a HFD and were found to develop colon polyps at a frequency of 23.1% and 31.6%, respectively. None of the *Hnf1a^+/+^* mice developed polyps, regardless of the diet (Figure 2B). Among the mice that developed polyps, only 2 were female, while the other 15 mice were male (Figure 2C). Interestingly, the polyps in the 2 female mice were both > 3 mm and were located in the proximal colon. On the other hand, the polyps in the males were generally smaller, at 1–2 mm in size and located in the middle and distal colon.

Colon polyps in *Hnf1a^A98V/+^* and *Hnf1a^A98V/A98V^* mice at 6 and 12 months showed a loss of normal gland architecture, increased cellularity and increased Ki-67 staining; however, there was no change in HNF1A expression (Figure 2D and Supplemental Figure 1). Most mice steadily gained weight until 9–11 months of age when their weight plateaued. Female mice were slightly heavier than male mice. We found that mice on the HFD gained more weight as compared with those on normal chow (Figure 2E). Surprisingly, the male *Hnf1a^A98V/A98V^* mice experienced failure to thrive by 12 months, since their uptrending weight dropped acutely but this was not observed in the females. Additionally, all mice on a HFD exhibited lower overall survival compared with those on the CD by 12 months of age with the *Hnf1a^A98V/A98V^* mice showing the worst overall survival (Figure 2F).

### Hnf1a^A98V^ mice on a HFD show altered pathways in immune function and energy metabolism.

**Figure 3:**
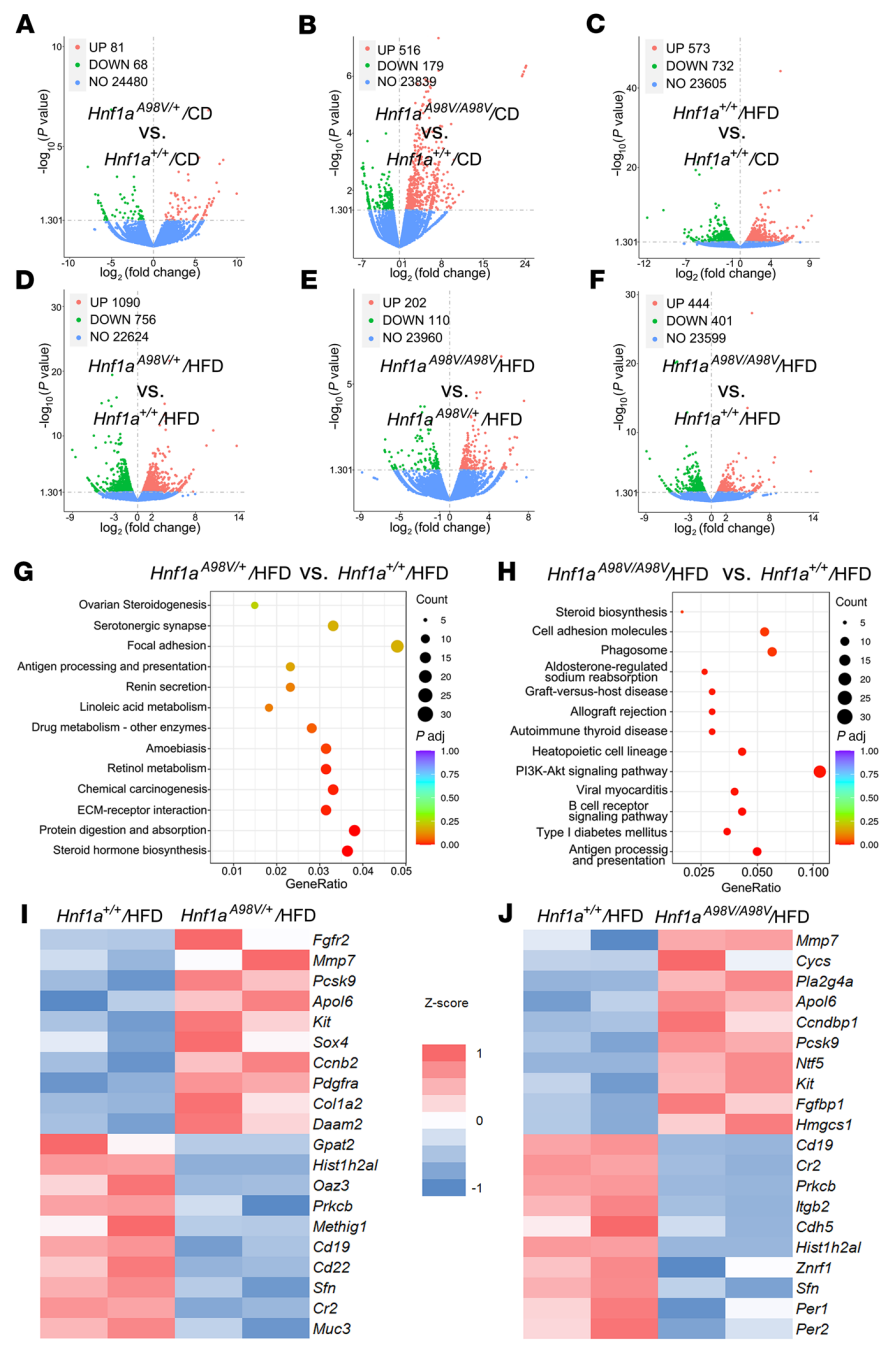
HNF1A^A98V^ with HFD showed altered pathways in immune function and energy metabolism. (**A**–**F**) Volcano plots of differential expression analysis for the indicated genotypes on control chow or HFD. (**G** and **H**) KEGG pathway enrichment of significant DEGs. (**I** and **J**) Heatmaps of cholesterol/lipid biosynthetic genes depicting representative differential gene expression between groups.

To define the transcriptomic landscape that allows polyps to develop, bulk RNA-Seq was performed using colon mucosal scrapings harvested from each mouse group. Volcano plots demonstrated differential gene expression as a function of the genotypes on the CD or HFD (Figure 3, A–F). There was a significant upregulation of genes in the *Hnf1a^A98V/A98V^* versus *Hnf1a^+/+^* mice on normal chow demonstrating that the *Hnf1a^A98V^* point mutation significantly altered the mouse transcriptome (Supplemental Figure 2 and Figure 3B). Simply changing the diet in *Hnf1a^+/+^* mice from a CD to a HFD also increased the number of differentially regulated genes (Figure 3C). On the HFD, differential gene expression was greatest in the *Hnf1a^A98V/+^* versus *Hnf1a^+/+^* mice and least when *Hnf1a^A98V/A98V^* mice were compared with the *Hnf1a^A98V/+^* (Figure 3, D and E). Differential expression when *Hnf1a^A98V/A98V^* mice were compared with the *Hnf1a^+/+^* mice showed intermediate changes (Figure 3F).

Since the polyps were discovered primarily in the *Hnf1a^A98V/+^*/HFD and *Hnf1a^A98V/A98V^*/HFD mice, KEGG analysis and representative genes in heatmaps were examined by comparing the different genotypes on the HFD (Figure 3, G and H). In general, genes relevant to lipid biosynthesis, diabetes, metabolism, and immune function were primarily affected (Figure 3, G and H, and Supplemental Figure 1). For instance, many fatty acid and cholesterol biosynthesis related genes were affected (17) including *pcsk9* (18)*,* *hmgcs1* (19), *mvk* (20), *pla2g4A* (20), *cycs* (17), and *cyp3a44* (21, 22). Importantly, *mmp7, daam2*, and *ccnb1/2* are β-catenin targets (23, 24) that increased in mice carrying 1 or both of the *Hnf1a* alleles on a HFD (Figure 3, I and J). The PI3K-Akt pathway regulates multiple cellular events including cell growth, proliferation, metabolism, protein and lipid synthesis, and autophagy (25), which were affected as well. Genes regulated by the PI3K-Akt pathway that were upregulated in the *Hnf1a* mutant mice include *pdgfra* (26, 27), *kit* (28), *hgf* (29), and *fgfr2* (30).

### Loss of function HNF1A^A98V^ variant activates the Wnt/β-catenin pathway in colon polyps.

**Figure 4:**
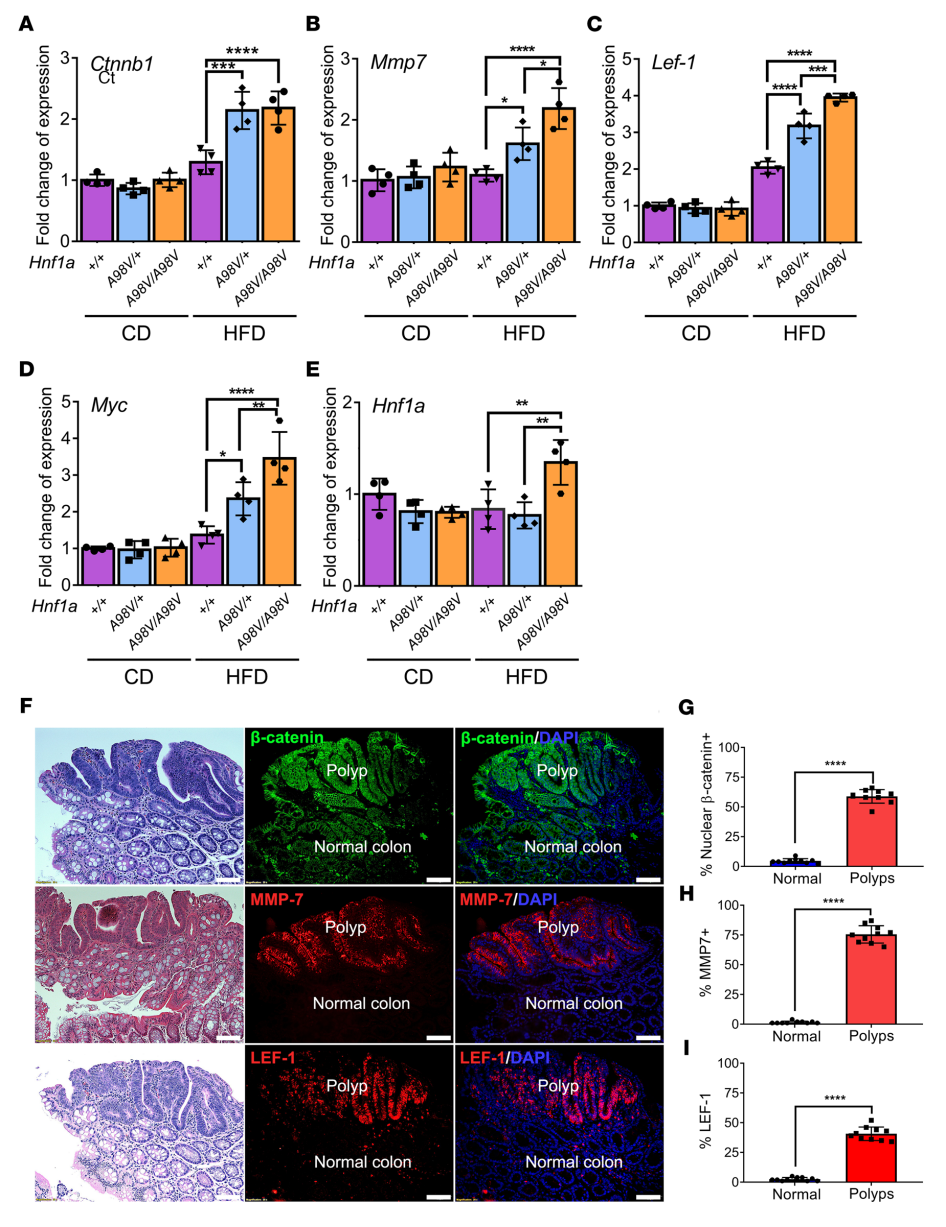
Loss of HNF1A function resulted in Wnt/β-catenin pathway activation and polyp formation. (**A**–**E**) The mucosal scrapings of the colon from each group were harvested and extracted for qPCR analysis (*n* = 4). The result was normalized to 18S ribosomal RNA. Statistical analysis was performed using 1-way ANOVA. Data represent mean ± SEM. *P < 0.05; **P < 0.01; ***P < 0.001; ****P < 0.0001. (**F**) Immunostaining of colon sections from mice with polyps was performed for β-catenin, MMP7, and LEF-1. The corresponding areas of polyp and normal colon were labeled. Scale bar: 50 μm. (**G**–**I**) The percentage of positive staining cells was calculated by averaging 10 random high-power field images from the polyps compared with the normal-appearing adjacent colon. The statistical analysis was performed using Student’s *t* test. Data represent mean ± SEM. ****P < 0.0001.

PI3K-Akt and Wnt/β-catenin pathways regulate overlapping gene targets, including *ccnd2, ntf5,* and *fgf2* (31). In addition, some focal and cell adhesion molecules are regulated by Wnt/β-catenin signaling (Figure 3) (32). For example, MMP7 is a known target gene of the Wnt pathway (23) and was elevated in both the *Hnf1a^A98V/+^* and *Hnf1a^A98V/A98V^* mice on a HFD compared with *Hnf1a^+/+^* mice, consistent with elevated Wnt/β-catenin and/or PI3K-Akt activity (Figure 3, I and J). Therefore, to assess whether β-catenin and other Wnt targets were elevated in these mice on a HFD, we performed quantitative PCR (qPCR) and found that *Lef-1* and *Myc*, in addition to *Ctnnb1* and *Mmp7*, were elevated in both groups in response to HFD compared with *Hnf1a^+/+^* mice (Figure 4, A–D). The mRNA levels showed no statistical difference in *Ctnn1b* expression among mice on the CD, regardless of the genotype. *Hnf1a* was only minimally elevated in the *Hnf1a^A98V/A98V^*/HFD colons, possibly related to a compensatory response to loss of HNF1A protein function (Figure 4E). In particular, immunofluorescence staining of the colons with polyps demonstrated that these Wnt targets increased only in the polyps (Figure 4, F–I). β-Catenin staining was more intense in the polyps, while β-catenin gene targets MMP-7 and LEF-1 increased only in the polyps (Figure 4, F–I).

The Wnt signaling pathway is a critical initiator in carcinogenesis in both hereditary and sporadic CRC (33). Previous studies have shown that Wnt/β-catenin signaling is aberrantly activated, with nuclear accumulation of β-catenin observed in 80% of CRC (34). The *Hnf1a^A98V/+^* and *Hnf1a^A98V/A98V^* colons showed elevated expression of Ki-67 in both the polyps and the normal adjacent mucosa (Supplemental Figure 1). In contrast, there was no significant change in HNF1A staining when comparing polyps and normal mucosa (Supplemental Figure 1). Next, we characterized the staining of the Wnt/β-catenin pathway. As shown in Figure 4F, the nuclear expression of β-catenin increased substantially in the polyps, while normal colon mucosa showed minimal nuclear β-catenin staining. Consistent with reverse-transcription PCR (RT-PCR) results, increased expression of MMP-7 and LEF-1 was observed, indicating upregulation of the Wnt/β-catenin pathway.

### Loss-of-function HNF1A^A98V^ variant activates Wnt/β-catenin pathway by reducing CDX2 expression.

**Figure 5:**
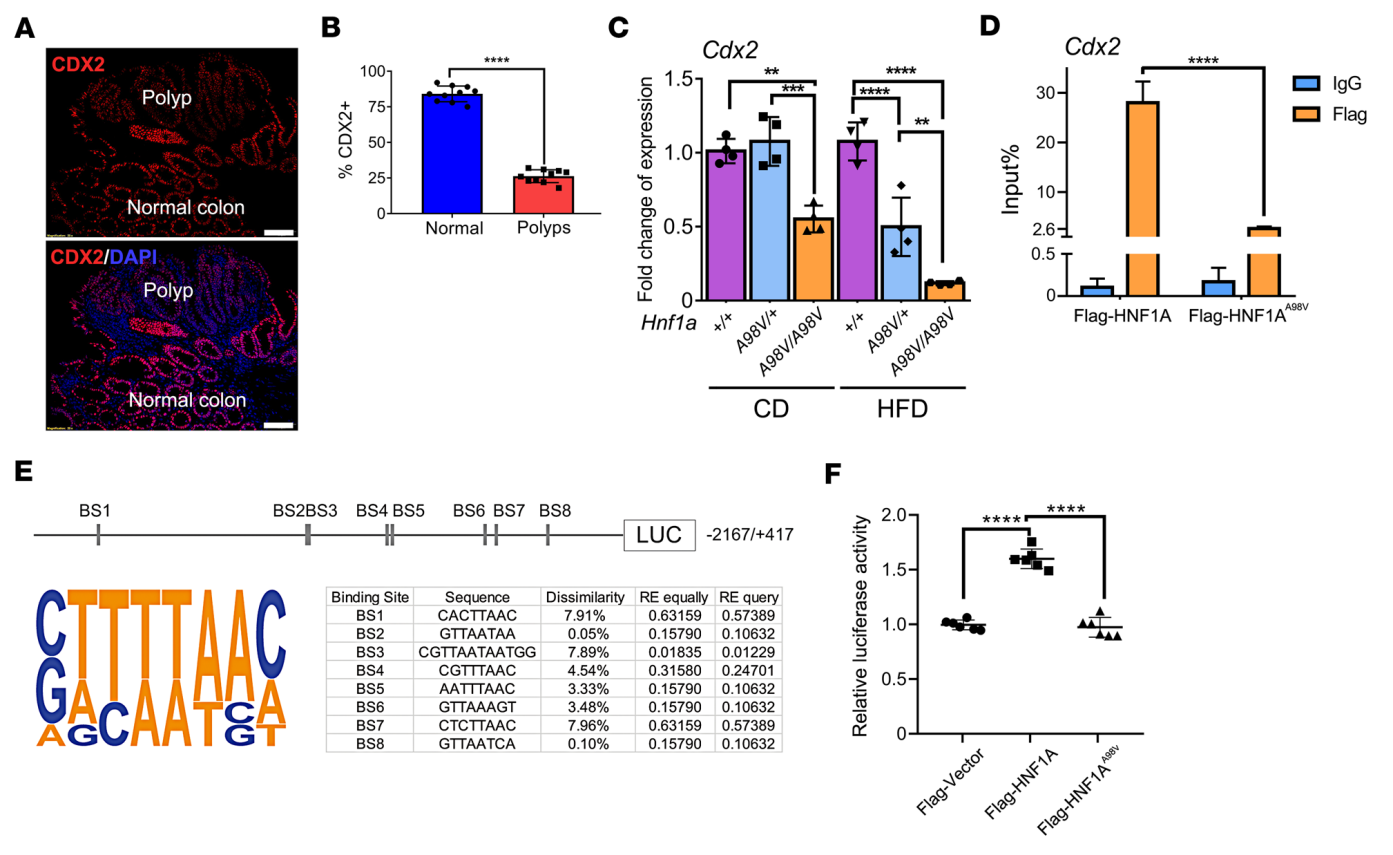
Loss of HNF1A function reduces CDX2 expression and results in activation of the Wnt/β-catenin pathway. (**A**) CDX2 expression in mouse colon polyps was evaluated by immunostaining. Scale bar: 50 μm. (**B**) The percentage of positive CDX2-stained cells was calculated by averaging 10 random high-power field images from the polyps compared with the normal-appearing adjacent colon. Student’s *t* test, ****P < 0.0001. (**C**) The mRNA levels of *Cdx2* were examined using qPCR in each mouse group. One-way ANOVA. **P < 0.01; ***P < 0.001; ****P < 0.0001. (**D**) The occupancy of HNF1A and HNF1A^A98V^ to the CDX2 gene was examined using ChIP analysis. Student’s *t* test, ****P < 0.0001. (**E**) The Cdx2 promoter luciferase construct. A total of 8 binding sites (BS) of HNF1A were predicted using the PROMO tool. (**F**) HT-29 cells were transfected with the Cdx2 promoter-Luc construct (–2,167/+417) and Flag-vector, Flag-HNF1A, or Flag-HNF1A^A98V^. The relative luciferase activity was determined using a dual-luciferase reporter assay system with a single-sample luminometer. The data represent 6 independent experiments. Data represent mean ± SEM. One-way ANOVA, ****P < 0.0001.

**Figure 6:**
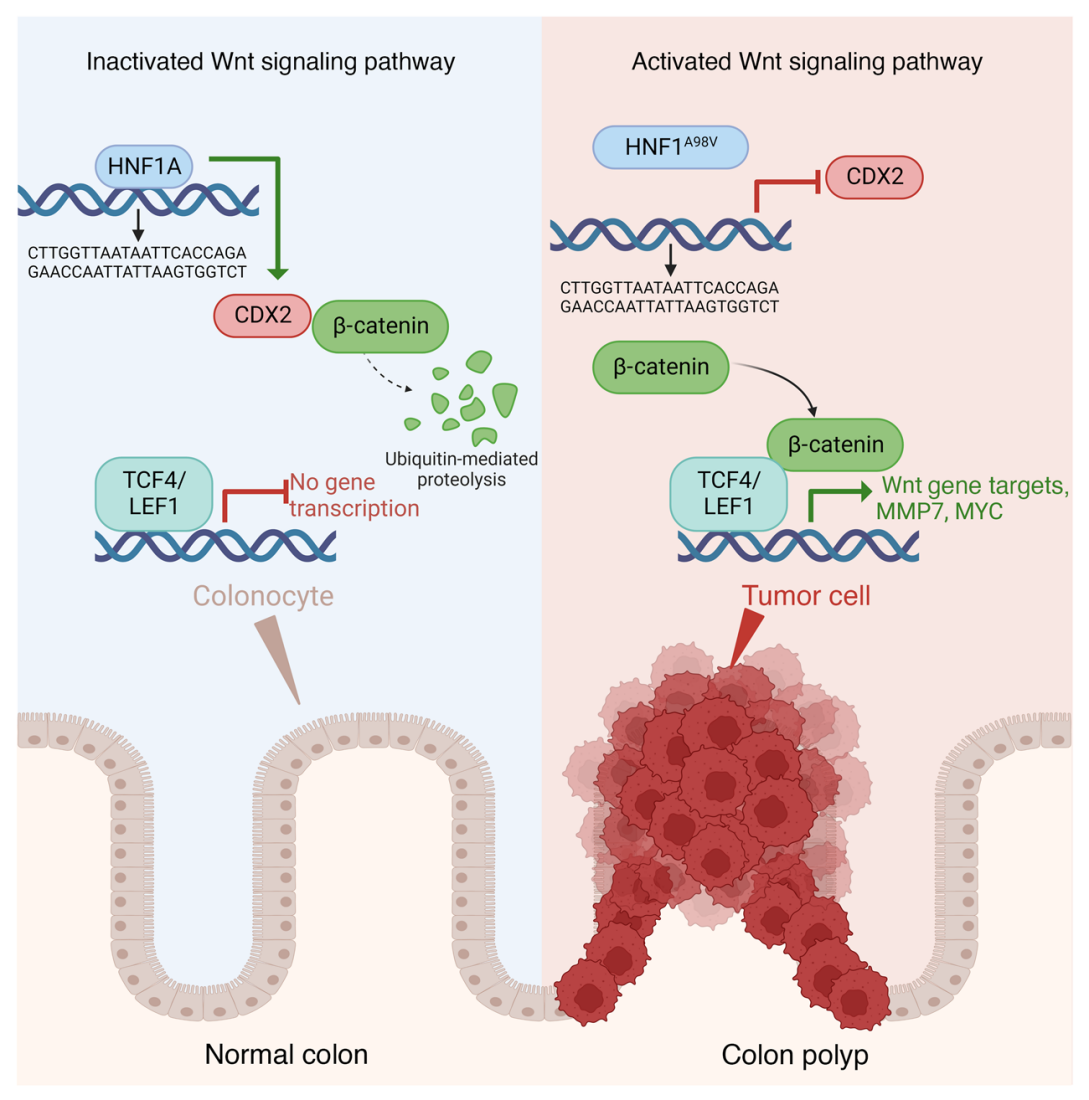
The role of HNF1A^A98V^ SNP in EO-CRC. In the colonocytes of normal mucosa (left), the HNF1A binds to the *CDX2* promoter. CDX2 binds β-catenin protein and disrupts its interaction with the DNA binding TCF factors, thereby silencing β-catenin/TCF target gene expression. *HNF1A^A98V^* is a missense mutation causing loss of protein function (right). This leads to a reduction of *CDX2* expression and subsequent activation of the Wnt/β-catenin pathway with a HFD, creating a landscape favoring colonic polyp formation.

To investigate how the HNF1A mutation led to increased β-catenin expression in the polyps, we first focused on the PI3K/AKT pathway, which showed a significant increase in *Hnf1a^A98V/A98V^*/HFD mice in our KEGG analysis (Figure 3H). Activation of genes in this pathway inhibits the cell-specification factor caudal-type homeobox transcription factor 2 (CDX2), which in turn can modulate the Wnt/β-catenin pathway (35). Wnt/β-catenin signaling normally maintains the proliferative stem cell compartment at the base of intestinal crypts (36). Previous studies have shown that CDX2 inhibits expression of Wnt target genes *LEF-1*, *Cyclin D1*, and *c-Myc*, suppressing cell proliferation (37). Additionally, CDX2 binds β-catenin protein and disrupts its interaction with the TCF family of DNA binding factors, which subsequently silences β-catenin/TCF gene targets (38, 39). Specifically, a highly conserved CDX2 subdomain in the N-terminus is critical for binding to β-catenin (39). Interestingly, previous studies in the intestine have analyzed *HNF1A* binding sites genome-wide using ChIP-Seq and show enrichment at the 10 kb downstream of the *CDX2* promoter (40, 41). Furthermore, HNF1A cooperates with other transcription factors to form a transcriptional regulatory network (10, 41). ChIP signals of FOXA2*,* CDX2, and HNF4A binding are enriched near *Hnf1* peaks with very similar patterns, indicating that these factors may interact as part of a single complex (40). Therefore, we investigated if there was decreased CDX2 expression in polyps from mice expressing both *Hnf1a* loss of function alleles. CDX2 staining in the polyps decreased compared with the normal mucosa (Figure 5, A and B). Similarly, RT-PCR revealed that HFD exacerbated depression of CDX2 mRNA in the *Hnf1a^A98V/+^* and *Hnf1a^A98V/A98V^* mice (Figure 5C). We performed a ChIP assay to determine whether HNF1A binds directly to CDX2 and evaluate whether this binding decreases with HNF1A^A98V^ mutation in HCT116 cells. Consistent with a prior study, we showed that HNF1A binds to CDX2 (40). This binding decreased significantly with HNF1A^A98V^ (Figure 5D). To illustrate if HNF1A transcriptionally increases CDX2 expression via binding to the CDX2 promoter, we used the CDX2 promoter Luciferase construct (–2,167/+417) (42). PROMO was applied to identify the putative transcription factor binding sites in the CDX2 promoter region (43, 44). A binding site for HNF1A was predicted at 1,204/1,215. Additionally, 7 potential binding sites were identified (Figure 5E). After cotransfection with the CDX2 promoter-Luc construct, Flag-HNF1A demonstrated a 1.5-fold increase in luciferase activity compared with overexpression of the empty vector, confirming that HNF1A binds to the CDX2 promoter. This increase was not observed in Flag-HNF1A^A98V^ (Figure 5F). Together, these data show that HNF1A binds to the CDX2 promoter and that loss of HNF1A function impaired CDX2 expression coincident with activation of Wnt/β-catenin signaling (Figure 6).

## Discussion

HNF1A is a transcription factor initially identified as a regulator of liver development. It is expressed in a variety of tissues and organs, including liver, pancreas, kidney, and intestine (45, 46). Numerous studies have shown that mutations in the *HNF1A* gene cause functional defects in islet β cells manifested by reduced insulin secretion, leading to MODY3 (11, 47–49). Here, we performed multigene panel NextGen sequencing on DNA from patients with EO-CRC and identified a missense mutation (A98V) located in the proximal DNA binding domain of HNF1A. Since the *HNF1A^A98V^* mutation decreases its DNA binding activity, we tested whether it was functional in vivo by introducing the variant into the mouse genome using CRISPR/Cas9 technology. Since the mice did not develop colon polyps on normal mouse chow (CD), we tested whether increasing the percentage of sugar or fat in the food would induce a phenotype. Accordingly, we found that both the *Hnf1a^A98V/+^* and *Hnf1a^A98V/A98V^* mice developed colonic polyps when fed a HFD, demonstrating that a loss of HNF1A function contributes to diet-related tumorigenesis.

According to the ClinVar database, a total of 812 variants of the HNF1A gene have been reported. The majority of mutations were single nucleotide (639/812). While prevalent, the importance of most single-nucleotide polymorphisms (SNP) remains unknown. Previous studies have identified A98V in the general population with a minor allele frequency of 2.7%, and it is associated with a slight increase in T2DM risk (50). While we present compelling evidence from our animal study that the combination of HNF1A^A98V^ and HFD results in colonic polyp development, these observations warrant further investigation into the broader implications on human CRC.

Prior epidemiologic studies show that metabolic disorders such as obesity and diabetes are associated with colon cancer (51, 52). Liu et al. reported a multivariate relative risk (RR) of 1.37 for EO-CRC in overweight (BMI, 25–29.9) women, RR of 1.93 in obese (BMI ≥ 30.0) women, compared with women with a normal BMI of 18.5 to 22.9 (46). Another study showed that being overweight or obese is associated with a 50% risk for colon cancer among both men and women (53). Similarly, the risk of CRC is ~27% higher in patients with T2DM than in nondiabetic controls. However, despite a well-established link between these metabolic disorders and colon cancer, the underlying mechanism is not understood. In our small cohort of *HNF1A^A98V^* participants with EO-CRC, 15% and 30% of these patients carried a diagnosis of DM and dyslipidemia/obesity, respectively. Collectively, the epidemiologic association of metabolic derangements (MAFLD) with colon cancer (54, 55) was a rationale for testing this association in our mouse model.

As others have observed (56), there were sex differences in the response to diet and colon cancer. Typically, male mice gain weight earlier compared with female mice while on a HFD. Additionally, male mice exhibit an increase in pancreatic β cell area as well as reduced insulin sensitivity after HFD feeding, whereas female mice do not (57, 58). Female mice are protected against HFD-induced metabolic changes (58), and ERβ-selective activation attenuates HFD-induced macrophage infiltration and epithelial cell proliferation in colon (59). In our study, homozygous male mice gained weight in a slower pace and reached a lower peak weight compared with their female counterparts while on a HFD. Previous research indicated that HNF1A-KO mice exhibit a phenotype resembling Laron-type dwarfism and non–insulin-dependent DM, likely due to a decrease in the expression of insulin-like growth factor I and lower insulin levels. This leads to stunted growth and elevated glucose levels in the bloodstream (60). However, this explanation fails to account for the absence of a similar weight gain pattern in female homozygous mice. This suggests the presence of sexual dimorphism but lacks sufficient data to draw firm conclusions.

In our study, we hypothesized that female sex hormones likely play a protective role against the reduced function of HNF1A A98V, leading to normal weight gain and fewer polyps compared with their male counterparts. This is supported by the finding that the polyps developed primarily in male mice (15 in male versus 2 in female), suggesting a potential protective effect of estrogen. Moreover, human studies have shown that sexual dimorphism exists at multiple levels in CRC. First, the age-standardized incidence of CRC rate in men is 45% higher compared with women according to the Global Cancer Observatory (61, 62). Second, women at a younger age are less likely to die from CRC than age-matched male participants (62, 63). Third, women show a higher frequency of right-sided (proximal) tumors and v-Raf murine sarcoma viral oncogene homolog B1 (BRAF) mutations (64, 65). Consistently, we noted a predominant distribution of mid and distal colon polyps in male mice (all 15 polyps), compared with a proximal location and large size polyps in female mice (all 2 polyps) in our study. While our clinical data do not show a sex difference in the 13 patients presenting with the HNF1A A98V mutation, it is likely that the number of patients included in this study is too small to reveal a sex difference. The mechanism of the sex-dependent differences is intriguing and warrants further investigation.

Current studies suggest that modifiable lifestyle factors, such as diet composition, play important roles in the occurrence and progression of CRC (66). A multicenter prospective cohort study on dietary patterns and risk of early-onset high-risk adenomas (EO-HRA) as precursors to EO-CRC reveals that Western diet consumption is an EO-HRA risk factor (OR, 1.67), while a “prudent diet” is protective (OR, 0.69) (67). Western dietary patterns were defined as high in red and processed meat, sugary drinks, refined grains, and desserts, while a prudent diet was characterized by a higher intake of vegetables, fruits, legumes, whole grains, and fish (68). A population-based case–control study in Ontario, Canada, identified greater consumption of sugary drinks (≥ 7 versus < 1 drinks/week; OR, 2.99; 95% CI, 1.57–5.68), and a more Westernized dietary pattern (quartile 4 versus 1; OR, 1.92; 95% CI, 1.01–3.66) as risk factors for EO-CRC (69). Furthermore, a recent mouse study indicates that a HFD drives colorectal tumorigenesis by inducing gut microbial dysbiosis, which is metabolic dysregulation with elevated lysophosphatidic acid that reduces gut barrier function (70). Although we tested 3 different diet compositions, polyps were induced to a greater extent in mice on a HFD than on a HSD or CD, respectively. The near absence of polyps on the HSD is likely due to the mode of administration, since it has been reported that sugar in the water stimulates fatty liver and metabolic derangements in mice; however, this only occurs when the sugar is placed in the water and not in the food (71, 72).

The Wnt/APC/β-catenin pathway is a critical initiator of both hereditary and sporadic CRCs. Mutations in the β-catenin, APC, AXIN1, and AXIN2 genes, or other Wnt pathway genes, can lead to translocation of the β-catenin protein into the nucleus, where it acts as a transcriptional activator by binding to T cell factors (TCFs) or lymphoid enhancer factor (LEF) family members and induces target genes such as *c-myc* and *cyclin D1* in CRC (73–76). In our study, we found that β-catenin levels increased concurrently with its pro-proliferative targets MYC and MMP-7 in the *Hnf1^A98V^* polyps, while CDX2 levels were depressed in the colonic polyps but not in the adjacent tissue. CDX2 is an important regulator of intestinal development and a biomarker of mature colon epithelial tissues (77, 78). Recent studies have demonstrated that it plays an essential role in tumorigenesis and has prognostic value in CRC (79, 80). CRC with reduced CDX2 expression is associated with an increased likelihood of aggressive features such as advanced stage, poor differentiation, vascular invasion, BRAF mutation, and the CpG island methylator phenotype (CIMP) (81). HNF1A binds to the *CDX2* promoter, while CDX2 binds β-catenin protein and disrupt its interaction with TCF factors preventing β-catenin derepression of TCF target genes (39). An additional role might be related to the ability of HNF1A (aka TCF1) to directly bind to β-catenin protein a its function (82).

In conclusion, we show that the HNF1A^A98V^ is a loss-of-function missense mutation prevalent in patients with EO-CRC that contributes to tumorigenesis under conditions of a HFD by reducing transcriptional induction of the *CDX2* gene. Collectively, our study unveils a role for the *HNF1A^A98V^* variant, a gene locus that lies at the intersection of the metabolic syndrome and CRC.

## Methods

### Patient blood samples and analysis.

The blood samples from patients with EO-CRC were analyzed by the University of Michigan (Ann Arbor, Michigan, USA) DNA sequencing core using the Qiagen gene array panel. Next-generation exon sequencing was performed with 158 patients’ blood samples.

### Plasmids and cell transfections.

Flag-tagged HNF1A and HNF1A^A98V^ were created by PCR amplification of human cDNA purchased from Genscript Biotech Corp. HCT116 cells (ATCC, CCL-247) were purchased from the EMSR Laboratory, University of Arizona Cancer Center, and grown in McCoy’s 5A medium (Thermo Fisher Scientific) supplemented with heat-inactivated 10% FBS and 100 U/mL penicillin and 100 μg/mL streptomycin (Thermo Fisher Scientific). Cells were cultured in atmospheric air enriched with 5% CO_2_ at 37°C. Cells were transfected with the HNF1A and HNF1A^A98V^ mutant using Lipofectamine 3000 (Invitrogen) and harvested after 48 hours. After collecting the cells from the plate, nuclear and cytoplasmic fractions were prepared using commercial the NE-PER (Thermo Fisher Scientific).

### Western blots.

Cell lysates or fractions were resolved on Invitrogen Novex 4%–20%, Tris-Glycine, 1.0 mm, Mini Protein Gels and transferred to nylon membranes for blotting with antibodies for HNF1A (Abcam, 204306), GAPDH (Cell Signaling Technology, 5147), FLAG (Sigma, F1804), and histone H3 (Cell Signaling Technology, 4499). After treating with HRP-conjugated secondary Ab (GE Healthcare), the complexes were visualized using Super Signal West Pico chemiluminescent substrate (Thermo Fisher Scientific).

### EMSA.

Double-strand DNA (5′-CTTGGTTAATAATTCACCAGA-3′) was purchased from Integrated DNA Technologies (IDT) and labeled with biotin following the manufacturer’s instruction (Pierce Biotin 3′ End DNA Labeling Kit). The binding reactions were carried out using the LightShift Chemiluminescent EMSA Kit (Thermo Fisher Scientific, 20148) and detected using the Chemiluminescent Nucleic Acid Detection Module Kit (Thermo Fisher Scientific, 89880).

### Animal studies.

*HNF1A^A98V^* transgenic mice were generated by the University of Michigan Transgenic Core using Crisper/Cas9 technology and then bred onto a homogenous C57BL/6J genetic background (The Jackson Laboratory). After weaning around 4 weeks of age, mice were fed 3 different diets, including a normal chow (CD; Envigo, 7013), a HFD (Envigo, 06414), and a HSD (Envigo, 86489) or a HSD composed of fructose (Envigo). Blood and organs were harvested, and body weight was measured. Survival was determined using Kaplan-Meier survival curves (GraphPad Prism 8).

### RNA-Seq.

RNA was extracted from the colonic mucosal scrapings using the Purelink RNA Extraction kit (Invitrogen) and was then submitted for RNA-Seq by Novogene. Fragments per kilobase of transcript sequence per millions base pairs (FPKM) sequenced were analyzed for differential gene expression, principal component, and Gene Ontology (GO) pathway enrichment analysis.

### Immunofluorescence.

Mouse tissues were fixed overnight at 4°C in 4% paraformaldehyde. Tissues were paraffin embedded, cut into 5 μm sections, and placed on frosted glass slides. Immunofluorescence staining was performed as previously described (83) using Ki-67 (Abcam 1:200), HNF1A (1:200), MMP7 (CST 3801 1:100), LEF-1 (CST 2230 1:100), CDX2 (Abcam 76541 1:400), and β-catenin (CST 37447 1:400). The percentage of positive cells was quantified as previously described (84) and was then plotted using GraphPad Prism 8.

### qPCR.

Scrapings of the mouse colonic mucosa were homogenized with a rotor-stator homogenizer in RNA lysis buffer containing 1% β-mercaptoethanol. RNA was isolated using the Purelink RNA Extraction kit (Invitrogen) following manufacturer’s instructions. cDNA was prepared by the iScript Reverse Transcription Supermix for qPCR using 1 μg RNA and diluted by 80 μL nuclease-free water. qPCR was performed using PowerUp SYBR Green Master Mix (Invitrogen) with 20 ng cDNA added to each reaction. Predesigned forward and reverse primer sets were purchased from Integrated DNA Technologies (IDT) and used at a final 500 nM concentration. qPCR was performed using the QuantStudio3 Real-Time PCR System (Applied Biosystems) with the following cycling conditions: 2 minutes at 50°C, 2 minutes at 95°C, denaturing step for 1 second at 95°C, extension and annealing for 1 minute at 60°C, and a dissociation melt curve stage to confirm primer specificity. The results are expressed as fold-increase mRNA expression of the gene of interest normalized to 18S expression by the ΔΔCt method.

### ChIP.

HCT116 cells were transfected with the Flag-HNF1A or Flag-HNF1A^A98V^ mutant using Lipofectamine 3000 (Invitrogen) and prepared for ChIP after 48 hours. EZ-Magna ChIP HiSens Kit from MilliporeSigma was applied. The immunoprecipitated purified DNA was used to amplify the Cdx2 gene using SYBR GREEN-based (Thermo Fisher Scientific) RT-PCR analysis. The following primers are used (40): Cdx2 forward 5′-GAAGTCAAGGGCAGTGGAAC-3′, reverse 5′-CCTCTGGGCTTCCATGATTA-3′. Data were represented as relative enrichment with respect to IgG control based on 2^−ΔΔCT^ method.

### Luciferase assay.

The CDX2 (–2,167/+417)-pGL3 basic-luciferase construct (42) was generated by Genecopoeia. HT-29 cells were transfected with CDX2 promoter-luciferase construct using Lipofectamine 3000 (Invitrogen), together with Flag-vector, Flag-HNF1A, or Flag-HNF1A^A98V^ mutant as described before. To normalize transfection efficiency, cells were simultaneously cotransfected with a pRLTK vector expressing the Renilla luciferase enzyme (pRL, Promega). After 48 hours, the luciferase activity was measured using the Dual-Luciferase Reporter Assay System (Promega).

### Statistics.

Results are shown as the mean ± SEM of 3 independent experiments. Shapiro-Wilk test was used to test for normality distribution. If the significance value of the Shapiro-Wilk test was greater than 0.05, the data were considered normal. For comparison among multiple groups, 1-way ANOVA or 2-way ANOVA was used depending on the nature of comparison. When there were more than 1 continuous dependent variables, 1-way multivariate ANOVA (MANOVA) was used to determine whether there were any differences between independent groups. The nonparametric Kruskal-Wallis test followed by the Games-Howell post-hoc test was used for multiple comparisons not distributed normally. Statistical analyses were performed in SPSS v22.0 (IBM SPSS Statistics). A *P* value of less than 0.05 was considered statistically significant.

### Study approval.

Written informed consent was received prior to participation. Blood samples were collected in accordance with the University of Michigan IRB (HUM00043430). All animal experiments were approved by the University of Arizona IACUC.

## Author contributions

HS, CAT, EMS, and JLM designed research studies. HS, RAS, JMM, MJV, and SS performed the experiments. HS, SD, JZ, SZ, RP, EMS, and JLM analyzed the data. EK and EMS provided patients’ data. HS, JZ, and JLM wrote the manuscript. JLM supervised the study.

## Supplementary Material

Supplemental data

## Figures and Tables

**Table 1:**
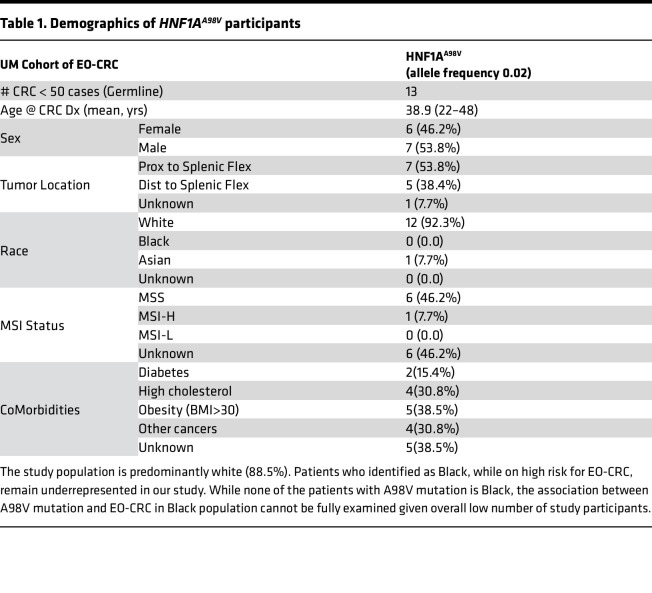
Demographics of *HNF1A^A98V^* participants

## Version 1 05/23/2023

In-Press Preview

## Version 2 07/10/2023

Electronic publication
