# Supplementary material for: High-fat diet plus HNF1A variant promotes polyps by activating β-catenin in early-onset colorectal cancer
Source: JCI Insight. 2023 Jul 10;8(13):e167163. doi: 10.1172/jci.insight.167163 (PMC10371337; doi:10.1172/jci.insight.167163)

Figure S1

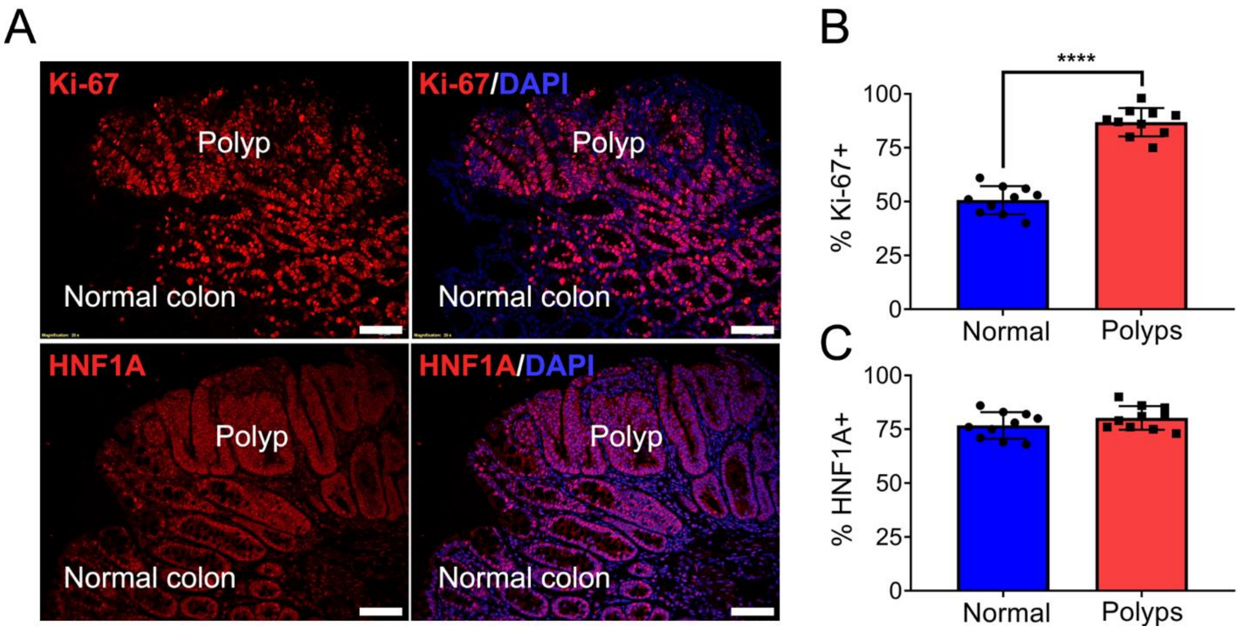

A. Representative IF images of Ki-67 and HNF1A staining in colon polyp and adjacent normal mucosa. Ki-67 (red); HNF1A (red); DAPI (blue). Scale bar = 100 μm. B-C. Percentage of positive staining cells were averaged by calculating 10 random high power field images. **** p<0.0001. Student t-test.

Figure S2

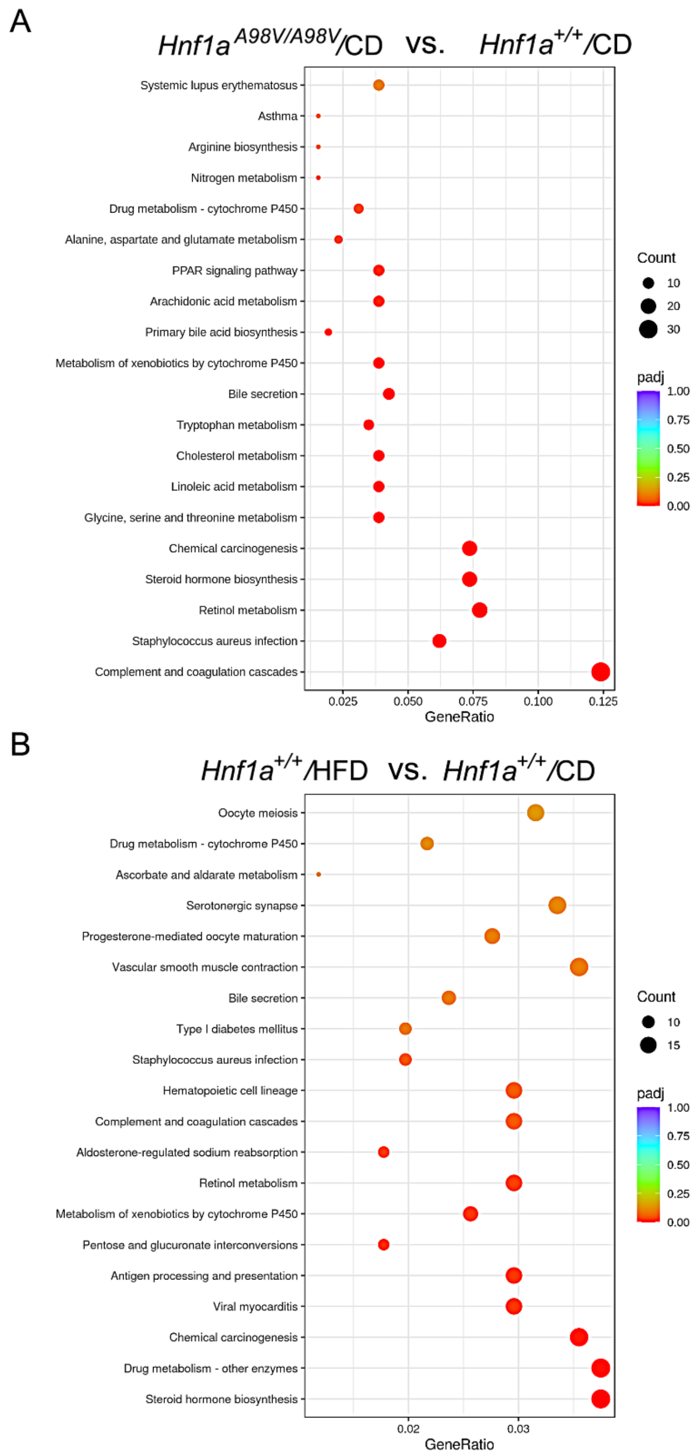

A. KEGG pathway enrichment of significant DEGs comparing *Hnf1a*$^{A98V/A98V}$ and *Hnf1a*$^{+/+}$ mice fed with CD. B. KEGG pathway enrichment of significant DEGs comparing the effect of diet in *Hnf1a*$^{+/+}$ (CD vs HFD).

Table S1. Demographics of EO-CRC Cases.

UM Cohort of Young Onset CRC	All Young CRC Cases	Cases Positive for Hereditary Cancer (germline mutations)	No Pathogenic variant identified in known cancer genes
	N=513	N=130	N=383
Sex:<br>Female<br>Male	 241 (47.0%)<br>272 (53.0%)	 66 (50.4%)<br>65 (49.6%)	 176 (46.1%)<br>206 (53.9%)
Mean age @ Dx (range)	39.9 (15-49)	37.2 (17-49)	40.9 (15-49)
Race:<br>White<br>African American<br>Asian<br>Other/Unknown	 454 (88.5%)<br>16 (3.1%)<br>16 (3.1%)<br>27 (5.3%)	 120 (92.4%)<br>0 (0.0%)<br>4 (3.0%)<br>5 (4.6%)	 333 (86.9%)<br>16 (4.2%)<br>12 (3.1%)<br>22 (5.8%)
Co-Morbidities:<br>Diabetes<br>High Cholesterol<br>Obesity	 60 (11.7%)<br>105 (20.5%)<br>128 (25.0%)	 12 (9.2%)<br>25 (19.1%)<br>36 (27.5%)	 48 (12.6%)<br>80 (20.9%)<br>92 (24.1%)

Table S2. HNF1A^{A98V} mice susceptible to colon polyps on HFD.

Control Diet	HNF1A$^{+/+}$	HNF1A$^{A98V/+}$	HNF1A$^{A98V/A98V}$	
# of Mice w/Polyps<br>6 mos	0/5	0/7	0/6	
9 mos		1/12		
12 mos	0/29	0/20	0/22	
	0/34	1/39	0/28	1/101
**High Fat Diet**	**HNF1A$^{+/+}$**	**HNF1A$^{A98V/+}$**	**HNF1A$^{A98V/A98V}$**	
6 mos	0/5	2/5	3/5	
8-9 mos	0/6	2/9	---	
12 mos	0/10	5/25	3/14	
14 mos	0/1	4/14	2/4	
	0/22	9/39	6/19	15/80=18.8%
**High Sugar Diet**	**HNF1A$^{+/+}$**	**HNF1A$^{A98V/+}$**	**HNF1A$^{A98V/A98V}$**	
6 mos		0/3	---	
8 mos		0/4	0/3	
9 mos		1/13	---	
12 mos	0/5	0/2	0/9	
	0/5	1/22	0/12	1/39
	0/61	11/100= 11%	6/59= 10.2%	

The mice of each genotype were fed with either CD, HSD or HFD. The mice were euthanized at different ages to examine the presence of polyps. The mice with positive polyps were recorded in each group and marked as red in the table. The percentage of polyps development in each group was calculated at the conclusion of the study.

Full unedited gel images

Full unedited gel of Figure 1C

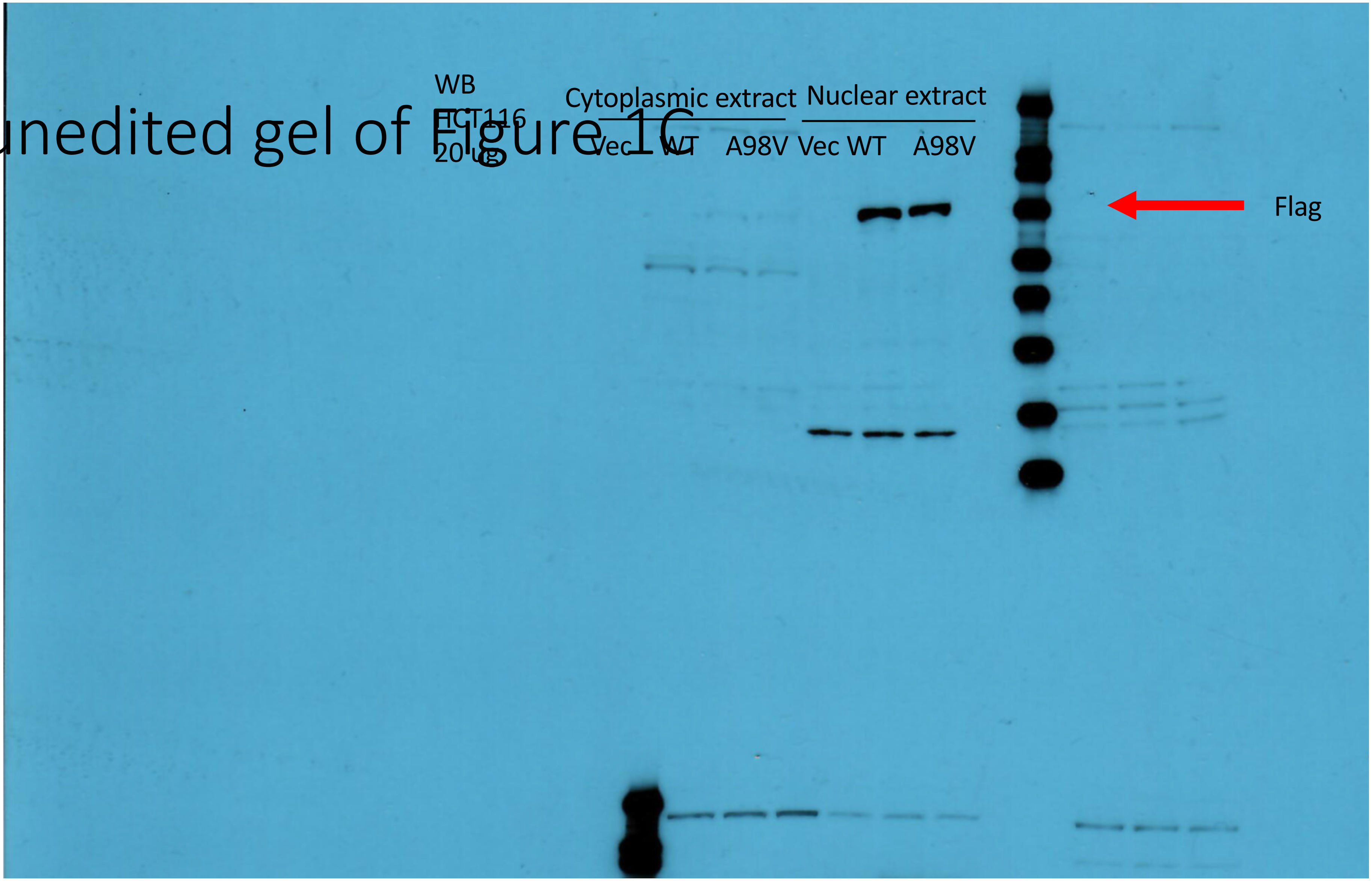

Full unedited gel of Figure 1C

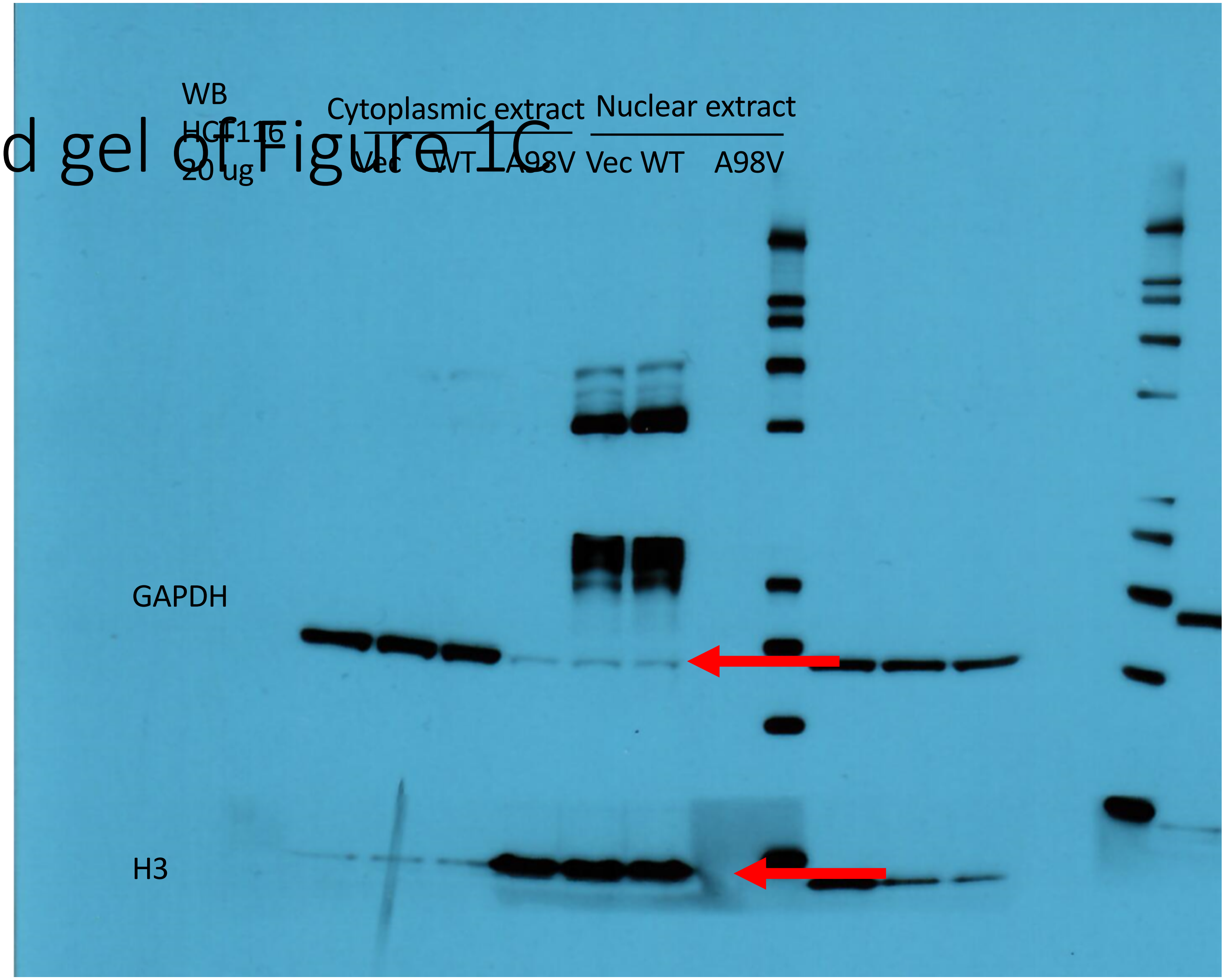

Full unedited gel of Figure 1D

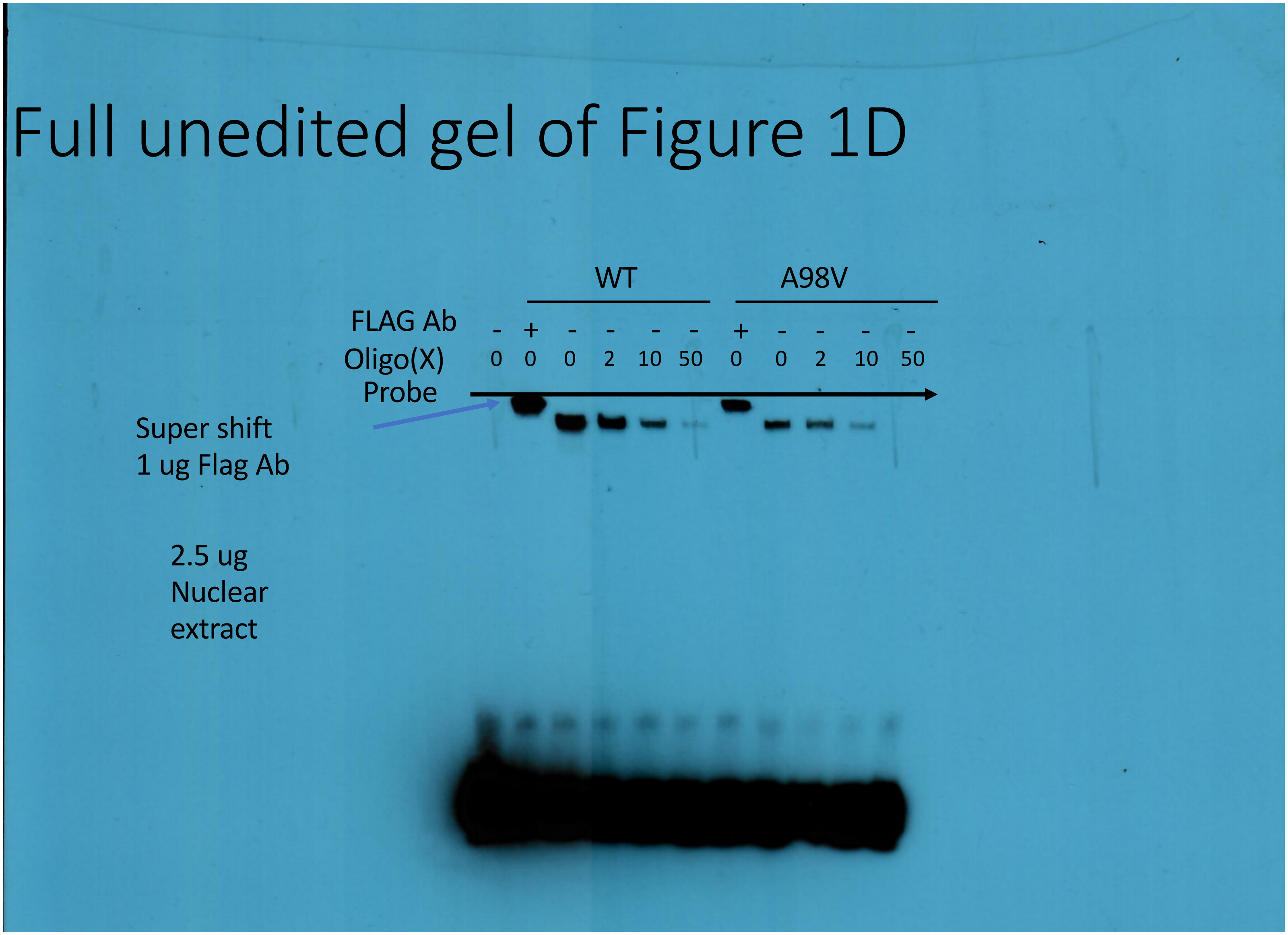

Supplement: Supplemental data [file jciinsight-8-167163-s051.pdf]
